# Optimizing the Context of Support to Improve Outcomes of Internet-Based Self-help in Individuals With Depressive Symptoms: Protocol for a Randomized Factorial Trial

**DOI:** 10.2196/21207

**Published:** 2021-02-02

**Authors:** Oliver Thomas Bur, Tobias Krieger, Steffen Moritz, Jan Philipp Klein, Thomas Berger

**Affiliations:** 1 Department of Clinical Psychology and Psychotherapy University of Bern Bern Switzerland; 2 Department of Psychiatry and Psychotherapy University Medical Center Hamburg-Eppendorf Hamburg Germany; 3 Department of Psychiatry and Psychotherapy Luebeck University Luebeck Germany

**Keywords:** depression, self-help, adherence, internet-based intervention, factorial design, problem-solving therapy, online, mental health, multiphase optimization strategy, digital health

## Abstract

**Background:**

Internet-based self-help interventions for individuals with depressive symptoms, in which the main component is often a web-based self-help program, have been shown to be efficacious in many controlled trials. However, there are also trials on self-help programs showing no significant effect when delivered in routine care, and some studies report high dropout and low adherence rates. Research suggests that these findings do not emerge primarily due to the specific content of a self-help program. It seems more important how a program is embedded in the context of human and automated support before and during the use of a self-help program.

**Objective:**

This study aims to better understand the effects of 4 supportive contextual factors on outcomes of and adherence to a web-based self-help program for depressive symptoms. In a factorial experiment, 2 of 4 supportive factors, for which there is evidence for their role on outcomes and adherence, are realized during the intervention—personal guidance and automated email reminders. The other 2 factors are realized before the intervention—a diagnostic interview and a preintervention module aimed at increasing the motivation to use the program with motivational interviewing techniques.

**Methods:**

The study is a full factorial randomized trial. Adults with mild to moderate depressive symptoms (Patient Health Questionnaire–9 score: 5-14) are recruited from the community through the internet and conventional media. All participants receive access to a web-based self-help program based on problem-solving therapy. They are randomized across 4 experimental factors, each reflecting the presence versus absence of a supportive factor (guidance, automated reminders, diagnostic interview, preintervention module) resulting in a 16-condition balanced factorial design. The primary outcome is depressive symptoms at 10 weeks post assessment. Secondary outcomes include adherence to the program, anxiety, stress, health-related quality of life, possible negative effects, and treatment satisfaction. Potential moderators and mediators (eg, treatment expectancy, problem-solving skills, working alliance with the study team) will also be investigated.

**Results:**

Ethical approval was received on January 20, 2020. The study was initiated in February 2020, and 240 participants have been enrolled in the study as of November 1, 2020. Recruitment for a total of 255 participants is ongoing. Data collection is expected to be completed by May 2021.

**Conclusions:**

A better understanding of relevant supportive factors in the dissemination of web-based interventions is necessary to improve outcomes of and adherence to web-based self-help programs. This study may inform health care systems and guide decisions to optimize the implementation context of web-based self-help programs for depressive symptoms.

**Trial Registration:**

ClinicalTrials.gov NCT04318236; https://clinicaltrials.gov/ct2/show/NCT04318236

**International Registered Report Identifier (IRRID):**

DERR1-10.2196/21207

## Introduction

Depression is one of the most common mental disorders that burdens society and individuals psychologically and financially [1,2]. Psychological consequences of depression include lower quality of life and more years lived with disability [3,4]. Although psychotherapy is an established evidence-based treatment option for depression [5], people often do not receive adequate care [6-8]. Internet-based self-help interventions are promising to reduce the burden of depression. During the last two decades, several research groups intensively studied the efficacy of internet-based self-help interventions and concluded that they effectively reduce depression [9-12].

Internet-based self-help interventions complement existing interventions in health care, addressing some of their limitations. Potential advantages of internet-based self-help interventions include that they are easily accessible, provide a high degree of anonymity, can be used independently of time and place, and can be provided to many people simultaneously. Hence, many authors suggest internet-based self-help interventions as a possibility to complement face-to-face psychotherapy to improve mental health care [13].

Although internet-based self-help interventions effectively reduce depressive symptoms, their potential might not be fully exploited. For example, studies [10] about internet-based self-help interventions for depression report a wide range of effect sizes (Hedges *g*=0.02-1.56). One study [14] that investigated widely used internet-based self-help interventions for depression failed to transfer the established effects into other settings, such as primary care. Further challenges of internet-based self-help interventions are low uptake rates (ie, logging into an intervention) and low levels of adherence (eg, completing modules of an intervention) [15,16].

One reason for diverging outcomes and adherence seems to be the degree of human support and guidance provided before and during the use of a self-help program. Current literature suggests that unguided internet interventions without human support at any stage tend to be associated with high dropout rates [17], lower adherence [18], and lower effects [11]. In a review [19], the authors suggested there were positive effects from guidance during the treatment on outcome in depressive patients. Additionally, several meta-analyses [9,12,20,21] report larger symptom reductions in guided self-help interventions with therapist support during the treatment compared to unguided self-help interventions without therapist support during the treatment. However, the differences between guided and unguided interventions may also be related to other factors such as the scope of diagnostic assessments or the length and content of a self-help program. These and other factors may confound the association between guidance, adherence, and outcomes. It is worth mentioning that in some studies [22,23] directly comparing self-help interventions with and without guidance, no significant differences were reported about the outcomes and number of modules completed.

In the review [19], the authors propose that other forms of human interaction (such as pretreatment contact) might also be beneficial for the treatment with internet-based self-help interventions. In a study [24] with patients that have social anxiety disorder, a diagnostic telephone interview conducted before an internet-based intervention significantly improved adherence to treatment and secondary outcomes of depression and stress.

Other aspects that potentially improve internet-based self-help intervention outcomes and adherence can be automated and realized without human contact. There is limited evidence that automated email reminders may improve adherence and outcomes of internet interventions. For example, a study [25] that compared semistandardized email feedback with fully standardized email feedback did not find a difference in the 2 conditions indicating that fully automated emails may be as effective as semistandardized feedback. Furthermore, in a transdiagnostic intervention, email reminders resulted in better outcomes for participants who had elevated co-occurring symptoms of anxiety and depression [26]. However, this did not apply to participants with elevated symptoms of either just anxiety or depression. In the same study [26], the reminders increased the number of people completing the intervention. Consistent with this finding, some participants mentioned that they experience email reminders helpful for adhering to the intervention [27].

Another possibility for increasing outcomes of and adherence to internet-based self-help interventions is to enhance the motivation of participants. A well-known method in face-to-face treatments to address ambivalence and enhance motivation is motivational interviewing [28]. High effect sizes and increased adherence were observed in a study [29] with motivational interviewing prior face-to-face psychotherapy treatment. A study [30] on an internet-based self-help intervention for social phobia was able to replicate these findings for internet-based self-help interventions to some extent—whereas participants of the group that received an additional motivational interviewing–based intervention did not show a higher magnitude of improvement, these participants were more likely to complete the treatment. Furthermore, for patients with depressive symptoms, a brief informational video about internet-based self-help interventions significantly increased the acceptance of internet-based self-help interventions [31].

Thus, several supportive contextual factors have been associated with better outcomes and increased adherence. Yet, it is not entirely clear which factors are crucial for a significant enhancement of internet-based self-help interventions. Consequently, clear guidelines for how to optimally embed internet-based self-help interventions into a context of supportive factors are missing. To fully exploit the potential of internet-based self-help interventions, dismantling studies are needed to understand how and which supportive factors are essential when disseminating internet-based self-help interventions. Often, studies that investigated the influence of a specific supportive factor such as guidance had other factors in their study design that potentially confounded the effect of guidance (eg, a diagnostic interview). Therefore, only conclusions about the whole treatment package (eg, internet-based self-help intervention, diagnostic interview, and guidance combined) and not about individual supportive factors (eg, either diagnostic interview or guidance) were possible. This entanglement limits insight into both the main effect of a given factor and possible interactions with other factors.

One reason for limited insight into essential supportive factors may be reliance upon RCTs in internet-based self-help intervention research. Although RCTs are the gold standard for establishing the efficacy or effectiveness of an intervention, they are not suited for investigating the effects of single supportive factors or specific treatment components. Because RCTs only compare the whole multifactorial intervention (treatment package) with another intervention or a control group, specific mechanisms are confounded with one another. Therefore, it is only possible to draw conclusions about the whole treatment package and not about the main and interactive effects of specific factors [32].

A new approach to getting more insight into how treatments work is the multiphase optimization strategy, which integrates perspectives, approaches, and concepts of various sciences [33]. Collins and Kugler [33] suggest that behavioral intervention research has focused too much on establishing the efficacy of treatments rather than understanding how treatments work and how they could be optimized. The multiphase optimization strategy's fundamental idea is to optimize interventions to meet specific criteria such as effectiveness, economy, or scalability. Interventions can be optimized by making decisions based on findings about which intervention components work and which intervention components do not work, which ones work well together, or which ones adversely affect each other.

The multiphase optimization strategy presents several experimental designs to optimize interventions. The most frequently used in behavioral sciences is the factorial design [34-36]. This design allows investigating multiple factors simultaneously within one trial. It can reveal which factors are active or inactive in influencing the desired outcomes. More specifically, factorial experiments allow exploring the main effects of and possible interactions between factors. Consequently, the findings of a factorial design study are suited to optimize a given intervention because they provide information about which factors can be kept and which factors can be omitted. Note that Collins and Kugler [33] do not claim that RCTs can be replaced with factorial designs. Rather, they suggest an integrative strategy that focuses both on optimizing interventions (for which there are better designs than RCTs) and establishing efficacy or superiority of interventions (for which RCTs are still the best option).

This study aims to further clarify the optimal context of support of internet-based self-help interventions for depressive symptoms. It uses a factorial design to test the impact of 4 factors and their combinations. These factors are (1) a diagnostic interview conducted before the intervention, (2) a preintervention module using techniques of motivational interviewing accessible before the intervention, (3) human guidance during the intervention, and (4) automated email reminders during the intervention.

## Methods

### Study Design

The study, including assessments and the self-help intervention, will be conducted online. Participants will not receive any financial reimbursement for taking part in the study. The study consists of a full factorial trial that includes 4 experimental factors. Each factor will be evaluated at 2 levels (either present or absent), resulting in a 16-condition (2×2×2×2) balanced full factorial design (Table 1). Factorial designs allow for reliably estimating all main effects and 2-factor interactions. To do so, the full sample (ie, participants from all 16 conditions) are used. Thereby, power remains associated with all participants as half of the participants are in a condition with a specific factor active, and half of the participants are in a condition with a specific factor inactive. This makes the factorial design efficient with respect to sample size and power.

**Table 1:** Overview of the 16 experimental conditions of the full factorial design—every factor is balanced; therefore, each is present and absent an equal number of times.

Condition	Diagnostic interview	Preintervention motivational interviewing module	Guidance	Email reminders
1	✓^a^	✓	✓	✓
2	✓	✓	✓	—^b^
3	✓	✓	—	✓
4	✓	✓	—	—
5	✓	—	✓	✓
6	✓	—	✓	—
7	✓	—	—	✓
8	✓	—	—	—
9	—	✓	✓	✓
10	—	✓	✓	—
11	—	✓	—	✓
12	—	✓	—	—
13	—	—	✓	✓
14	—	—	✓	—
15	—	—	—	✓
16	—	—	—	—

^a^Factor is present.

^b^Factor is absent.

### Participant Eligibility

Eligible participants are German-speaking residents of Switzerland, Germany, Austria, and Lichtenstein. Inclusion criteria are (1) being at least 18 years of age; (2) meeting criteria for mild to moderate depression (score between 5 to 14 on the Patient Health Questionnaire–9) [37]; (3) providing written informed consent; (4) having access to the internet as well as an email account; and (5) providing an emergency contact before treatment. The study allows participants to take part even if they currently receive constant antidepressant medication or psychotherapy treatment. Exclusion criteria are (1) having a history of a psychotic or a bipolar disorder and (2) having increased suicidal tendencies (a score >7 on the Suicide Behaviors Questionnaire-Revised)[38,39].

### Study Procedure

Interested participants can leave an email address on our study website [40]. Participants will automatically receive study information and an informed consent sheet (by email). After providing informed consent, participants are invited to complete the baseline assessment. Study eligibility is assessed and if included in the study, participants must wait 2 weeks before they can start with the intervention. Depending on which condition participants are randomized to, during these 2 weeks, participants either wait, are diagnostically interviewed, receive access to the preintervention motivational interviewing module, or receive both the interview and the preintervention motivational interviewing module (see Table 2).

**Table 2:** Study flow and overview of study variables.

Study activity			Study period and timepoint											
			Allocation				Postallocation							Follow-up
			Week 0, T0		Week 0-2		Week 2, T1		Week 4, T2		Week 10, T3		Week 16, T4	
**Enrollment**														
	Registration	✓		—^a^		—		—		—		—		
	Informed consent	✓		—		—		—		—		—		
	Eligibility screening	✓		—		—		—		—		—		
	Randomization	✓		—		✓		—		—		—		
**Treatment**														
	Internet intervention	—		—		✓		✓		✓		✓		
**Factors**														
	Diagnostic interview	—		(✓)^b^		—		—		—		—		
	Motivational interviewing module	—		(✓)		—		—		—		—		
	Guidance	—		—		(✓)		(✓)		(✓)		—		
	Automated emails	—		—		(✓)		(✓)		(✓)		—		
**Surveys**														
	Patient Health Questionnaire–9^c^	✓		—		✓		✓		✓		✓		
	Generalized Anxiety Disorder–7	✓		—		—		—		✓		✓		
	Patient Health Questionnaire–Stress	✓		—		—		—		✓		✓		
	Short Form health survey–12	✓		—		—		—		✓		✓		
	Suicide Behaviors Questionnaire–Revised	✓		—		—		—		✓		✓		
	Social Problem-Solving Inventory–Revised	✓		—		—		—		✓		✓		
	Client Satisfaction Questionnaire	—		—		—		—		✓		—		
	Working Alliance Inventory for Guided Internet Interventions	—		—		—		✓		✓		—		
	Credibility/Expectancy Questionnaire	✓		—		✓		✓		—		—		
	Inventory for the Assessment of Negative Effects of Psychotherapy	—		—		—		—		✓		✓		
	System Usability Scale	—		—		—		—		✓		—		

^a^The study activity was not applied at this point.

^b^Parentheses indicate that factors apply to half of the participants.

^c^Primary outcome.

If individuals are excluded, they can make use of the intervention outside of the study. However, participants reporting suicidal ideation first need to confirm that they are in touch with their emergency contact or a psychotherapist. We offer to provide a contact for professional psychological help in case participants are severely depressed.

### Recruitment

Participants are recruited through depression-related websites, radio interviews, self-help groups, Facebook groups, Google ads, and the website of the University of Bern (Switzerland). The description of our study includes a link to the study website. Written informed consent to participate in the study is obtained from all participants.

### Intervention

The web-based self-help program *Herausforderungen meistern (overcoming challenges)* (HERMES) is based on problem-solving therapy [41]. The first, second, and last author developed the online program at the University of Bern. The problem-solving therapy intervention includes an introductory module and 3 toolkits: (1) Feeling, (2) Thinking, and (3) Acting. Problem-solving therapy shares various assumptions of cognitive behavioral therapy but focuses more explicitly on problems causing distress and problem-solving skills. We recommend that participants use the intervention approximately 1 hour per week and complete each module or toolkit within 2 weeks. This results in 8 weeks of recommended program use. An online problem-solving therapy intervention has previously been investigated in a 3-arm RCT [42]. Results indicated that, compared to a waiting list control group, the online problem-solving therapy intervention was as effective as an online cognitive behavioral therapy intervention in reducing symptoms of anxiety and depression [42].

Within the factorial design, 4 factors are realized. The first factor consists of a diagnostic telephone interview conducted before the self-help program. The second factor is a preintervention module based on motivational interviewing presented before the self-help program. The module aims at initiating a reflection process about one’s motivation for using the intervention [29]. The third factor is human support during the self-help program with personalized weekly emails. Guidance contains answering questions from participants within 3 working days and giving regular feedback on progress once a week. It is carried out by trained Master and PhD students who are supervised by licensed psychotherapists. The fourth factor is a set of weekly automatically sent emails during the self-help program. The emails inform participants on how far they should be in the program approximately, suggest content to work on next, and remind participants that they take part in a study. In contrast to human support (guidance), these emails are not individualized and contain the same information for all participants. In addition to these emails, prompts are sent to participants who have not logged in for 1 week. Our research focuses on investigating the context of human and automated support when providing web-based interventions. This implies that all participants receive the same main intervention with all program components of HERMES and that the main intervention is not changed throughout the whole study.

### Study Outcome Measures

All outcome measures will be assessed online with validated German versions of the original questionnaires.

#### Primary Outcome Measure

Symptoms of depression will be assessed with the self-reported measure Patient Health Questionnaire–9 [37]. The Patient Health Questionnaire–9 has good diagnostic validity, sensitivity, and specificity and is a commonly used measure to assess and monitor depression severity [43].

#### Secondary Outcome Measures

Adherence is defined as the extent to which participants use the intervention. Following the suggestion of Donkin et al [44], a composite score encompassing time spent in the intervention, number of modules completed, number of exercises completed, number of log-ins, and number of clicks in the intervention will be used to measure adherence to the intervention. The composite score will be created by averaging the *z* scores of these indicators. Furthermore, and for exploratory purposes, we will also run the analyses with each of these indicators of adherence. Symptoms of anxiety will be assessed with the Generalized Anxiety Disorder–7 [45]. Symptoms of stress will be assessed with the stress subscale of the Patient Health Questionnaire [45]. Health-related quality of life will be assessed with the Short Form Health Survey–12 [46,47]. Suicidal ideation will be assessed with the Suicide Behaviors Questionnaire–Revised [38,39]. Problem solving will be assessed with the Social Problem Solving Inventory-Revised [48,49].

### Treatment Characteristics

Possible adverse effects of the intervention will be assessed with the Inventory for the Assessment of Negative Effects of Psychotherapy [50]. Client satisfaction will be measured with the Client Satisfaction Questionnaire [51,52]. System usability will be assessed with the System Usability Scale [53,54].

### Moderators and Mediators

Demographic information about participants will be assessed at baseline. Treatment expectancy will be assessed with the Credibility/Expectancy Questionnaire [55]. Working alliance with the online coaches will be assessed with the Working Alliance Inventory for Guided Internet Interventions [56].

### Randomization

The online platform Qualtrics (Qualtrics XM) randomizes participants in 2 steps. First, after T0 and before any contact with the study team, participants are randomized automatically to 1 of 4 groups (1, diagnostic interview and motivational interviewing module; 2, diagnostic interview; 3, motivational interviewing module; 4, no factor). The first randomization is stratified (either mild or moderate depressive symptoms). Second, after 2 weeks and completing T1, participants are randomized to 1 of 4 groups (1, guidance and email reminders; 2, guidance; 3, email reminders; 4, no factor). Both times, block randomization ensures a balance in sample size across groups over time. A schedule of enrollment and participation is shown in Table 2.

### Data Collection, Management, and Analysis

Participants complete questionnaires at all 5 time points online via Qualtrics. We manually invite participants to complete the baseline questionnaire (T0). The 4 subsequent time points (after 2, 4, 10, and 16 weeks) are automatically triggered once T0 is completed. We try to limit the amount of missing data from survey attrition by reminding participants after 5 and 10 days to complete the questionnaires.

### Statistical Analysis

Statistical reporting will follow CONSORT [57] and CONSORT-EHEALTH standards [58]. We will conduct primary analyses using intention-to-treat. The primary outcome is the change in Patient Health Questionnaire–9 score from baseline to 10 weeks and 16 weeks. Dropout rates are examined per condition. Before the analysis, we will examine baseline predictors of attrition. If it appears that attrition is related to measured aspects of the participants, we will include those measures as covariates in the models.

To test for the main and interaction effects of treatment components on primary and secondary outcomes, linear mixed model analysis of variance will be used. This approach uses all available data on each subject and does not involve the substitution of missing values but estimates parameters about missing values. However, sensitivity analyses will explore the impact of the imputation of missing values before computing the mixed models. The main effects and interactions will be based on aggregates across experimental conditions. The purpose of the factorial experiment is not to compare the 16 conditions to each other but to estimate the main effects of the 4 factors and interactions between the factors. For example, the main effect of the diagnostic interview will be estimated by comparing the mean of the experimental conditions in which this factor is present (conditions 1-8 in Table 1) versus the mean of the experimental conditions in which this factor is not present (conditions 9-16 in Table 1). No adjustment for multiple testing will be applied in the estimation of statistical significance because, in the optimization phase of the multiphase optimization strategy framework, the emphasis is on deciding what components will make up the optimized intervention [33]. Only a future RCT can then establish the superiority of the optimized intervention over other conditions.

### Power Analysis

We conducted an a priori power analysis for small-to-medium effect sizes (Cohen *d*=.35) for main effects and interactions between 2 factors (eg, guidance and diagnostic interview) on change in depressive symptoms (G-Power 3.1). From a clinical perspective, smaller effects are considered to be less relevant [59]. For type I error α=.05, with a common power of .80 to detect effects. Based on previous studies, we assume that our measurements regarding pre, post, and follow-up correlate at approximately *r*=.60. For a factorial design, this signifies a sample of n=204 to detect effects. Because we expect a dropout rate of about 20%, the planned sample size is n=255. For every condition, roughly 15 participants are required.

## Results

The study was registered at ClinicalTrials.gov (NCT04318236). The ethics committee of the canton of Bern (*Kantonale Ethikkommission Bern*) approved the study on January 20, 2020 (2019-01795). Recruitment started in February 2020. As of November 1, 2020, out of 1480 interested individuals, 409 individuals have completed T0, and 240 participants have been enrolled in the study.

## Discussion

### Overview

The primary outcome is depressive symptoms 10 weeks after baseline. Several secondary outcomes will be measured, such as symptoms of anxiety and stress, health-related quality of life, suicidal ideation, and problem solving. Possible moderating (age, gender, and adherence) and mediating (treatment expectancy, therapeutic alliance) effects will be tested. Furthermore, negative effects of psychotherapy, treatment satisfaction, system usability, and dropout rates will also be measured and inspected. This study builds on a wealth of encouraging efficacy studies of internet-based self-help. It promises to provide a more detailed insight into which supportive context factors enhance outcomes of and adherence to internet-based self-help interventions for depressive symptoms. Furthermore, the study may also inform about possible mediation and moderation effects that could provide more information about how or why internet-based self-help interventions for depressive symptoms work.

### Strengths and Limitations

Our study has been designed to shed more light on the supportive context of internet-based self-help interventions. It deconstructs a treatment package and explores active and inactive supportive factors. Understanding which factors do and do not work will help us get closer to the goal of delivering internet-based self-help interventions optimally. According to the guidelines of multiphase optimization strategy, a future RCT should test an intervention providing an optimal supportive context based on our findings, against an intervention providing a context that is usual in studies about internet-based self-help interventions (eg, an intervention with guidance). With such a study, the possible superiority of the optimized context could be established.

Limitations of this study are comparable to those of the majority of studies about internet-based self-help interventions. The sample of this study is self-selected and participants become aware of our study through the internet. This limits the generalizability of possible findings to regular clinical settings or individuals that rarely use the internet.

### Conclusion

To improve outcomes to future internet-based self-help interventions for depression, this study could provide recommendations on how to optimize the context of human and automated support. Based on findings of active and inactive factors and the interactions thereof, recommendations could be made for future research and the implementation and dissemination of internet-based self-help interventions in routine care.

## Abbreviations

CONSORT: Consolidated Standards of Reporting Trials
CONSORT-EHEALTH: Consolidated Standards of Reporting Trials of Electronic and Mobile Health Applications and Online Telehealth
HERMES: Herausforderungen meistern (overcoming challenges)
RCT: randomized controlled trial
